# Data mining of an acoustic biomarker in tongue cancers and its clinical validation

**DOI:** 10.1002/cam4.3872

**Published:** 2021-05-02

**Authors:** Yudong Xiao, Tao Wang, Wei Deng, Le Yang, Bin Zeng, Xiaomei Lao, Sien Zhang, Xiangqi Liu, Daiqiao Ouyang, Guiqing Liao, Yujie Liang

**Affiliations:** ^1^ Department of Oral and Maxillofacial Surgery Guanghua School of Stomatology Guangdong Provincial Key Laboratory of Stomatology Sun Yat‐sen University Guangzhou China

**Keywords:** acoustic analysis, diagnostic tests, quality of life, speech biomarker, support vector machine, tongue cancer

## Abstract

The promise of speech disorders as biomarkers in clinical examination has been identified in a broad spectrum of neurodegenerative diseases. However, to the best of our knowledge, a validated acoustic marker with established discriminative and evaluative properties has not yet been developed for oral tongue cancers. Here we cross‐sectionally collected a screening dataset that included acoustic parameters extracted from 3 sustained vowels /ɑ/, /i/, /u/ and binary perceptual outcomes from 12 consonant‐vowel syllables. We used a support vector machine with linear kernel function within this dataset to identify the formant centralization ratio (FCR) as a dominant predictor of different perceptual outcomes across gender and syllable. The Acoustic analysis, Perceptual evaluation and Quality of Life assessment (APeQoL) was used to validate the FCR in 33 patients with primary resectable oral tongue cancers. Measurements were taken before (pre‐op) and four to six weeks after (post‐op) surgery. The speech handicap index (SHI), a speech‐specific questionnaire, was also administrated at these time points. Pre‐op correlation analysis within the APeQoL revealed overall consistency and a strong correlation between FCR and SHI scores. FCRs also increased significantly with increasing T classification pre‐operatively, especially for women. Longitudinally, the main effects of T classification, the extent of resection, and their interaction effects with time (pre‐op vs. post‐op) on FCRs were all significant. For pre‐operative FCR, after merging the two datasets, a cut‐off value of 0.970 produced an AUC of 0.861 (95% confidence interval: 0.785–0.938) for T_3‐4_ patients. In sum, this study determined that FCR is an acoustic marker with the potential to detect disease and related speech function in oral tongue cancers. These are preliminary findings that need to be replicated in longitudinal studies and/or larger cohorts.

## Graphical abstract

Formant centralization Ratio was primarily approved a sensitive acoustic parameter to imply the general articulation function in patients with oral tongue cancer by support vector machine. Clinical validation in the context of preoperative analysis and longitudinal follow‐up further approved its discriminative and evaluative properties, which need to be reproduced in longitudinal studies with larger cohorts.

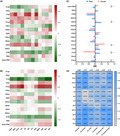

## 1 INTRODUCTION

Tongue cancer is the most common subtype of oral cancer.^1^, ^2^ It affects the tongue, which is the most important anatomical structure for speech utterance.^3^ Theoretically, any structural defects or functional impairments within the tongue body may cause changes in speech. In terms of resonance and articulation, these changes are typically embodied in vowel formant frequencies and acoustically perceived speech sounds.^4^ Such speech indexes, in turn, carry abundant information about disease status.^5^ Ideally, speech data are capable of indicating thorough details about the lesions, including the location, size, and degree of invasion. Therefore, the identification of a characteristic speech biomarker for tongue cancers is of clinical importance and may provide a convenient pathway to quantify the speech function.

Speech biomarkers have been widely reported in disease discrimination among a broad spectrum of diseases or disorders such as Parkinson's disease,^6^, ^7^ autism spectrum disorder,^8^ primary progressive aphasia,^9^ apraxia of speech,^10^ and emotional status.^11^ However, the common causes of these speech disorders share no structural changes, but rather, neurologic ailments. On the contrary, head and neck cancers are characterized by structural lesions, and different subsites take variant effects on speech function.^12^ For example, laryngeal diseases commonly manifest as a voice handicap,^13^ while tongue cancers may present as articulation disorders.^14^ The anatomical region of tongue cancers may also cause differing patterns of articulation disorders.^15^ Thus, a preliminary study of speech biomarkers for tongue cancers should be restricted to a specific region of the tongue (e.g., to lesions located on the lateral mobile tongue) to guarantee the homogeneity of subjects.

The identification of pertinent feature sets that underlie the nature of the disease is critical to the effectiveness of a speech biomarker. Therefore, selecting proper acoustic features is of utmost importance. Abundant features of different physiological or psychological interpretations can be extracted based on acoustic, spectral, and cepstral measures from the speech signal.^5^ Acoustic features typically include fundamental frequency (F0) and formant frequencies. The vocal folds within the larynx vibrate to produce the F0 and corresponding harmonics that are perceived as voice pitch, whereas formants are the resonant frequencies of the vocal tract.^16^ Given that tongue cancers mainly affect vocal resonance via tongue position embedded in speech dynamics, we predefine a set of potential acoustic features according to the review of Kent et al.^17^ (see Table S1).

Not all tongue cancers manifest in speech impairments.^12^ There is a distant projection from acoustic features to disease status, wherein an intermediate should be established to bridge the gap. As mentioned previously, predefined feature sets are chosen to reflect tongue position embedded in speech dynamics. Specifically, the production of consonant phonemes implicates maximal information about speech dynamics.^18^ Therefore, we selected consonants that reflect tongue mobility and vowels to make consonant–vowel (CV) syllables to bridge acoustic features and disease status (see Table S2).

Thus, the present study investigated which acoustic feature could be used as a speech biomarker with both linguistic and clinical implications, using a two‐tier approach. During the first stage of discovery and linguistic identification, we collected a dataset including the acoustic parameters extracted from 3 sustained vowels /ɑ/, /i/, /u/ and binary perceptual outcomes from 12 CV syllables. Second, we validated the pre‐operative and peri‐operative clinical efficacy of the speech biomarker with regard to disease status, treatment modality, and speech‐related quality of life (QoL), as it has been shown that speech impairments in tongue cancers significantly worsen QoL.^19^ Finally, we used an outcome measurements triad that combined Acoustic analysis, Perceptual evaluation and QoL assessment, herein referred as APeQoL.

## 2 MATERIALS AND METHODS

All procedures performed in this study involving human participants were in accordance with the ethical standards of the institutional and national research committee and with the 1964 Helsinki declaration and its later amendments or comparable ethical standards. The protocol was approved by the Ethical Committee of Affiliated Hospital of Stomatology at Sun Yat‐Sen University. Written informed consent was obtained from each participant.

### 2.1 Dataset for marker screening

**FIGURE 1:**
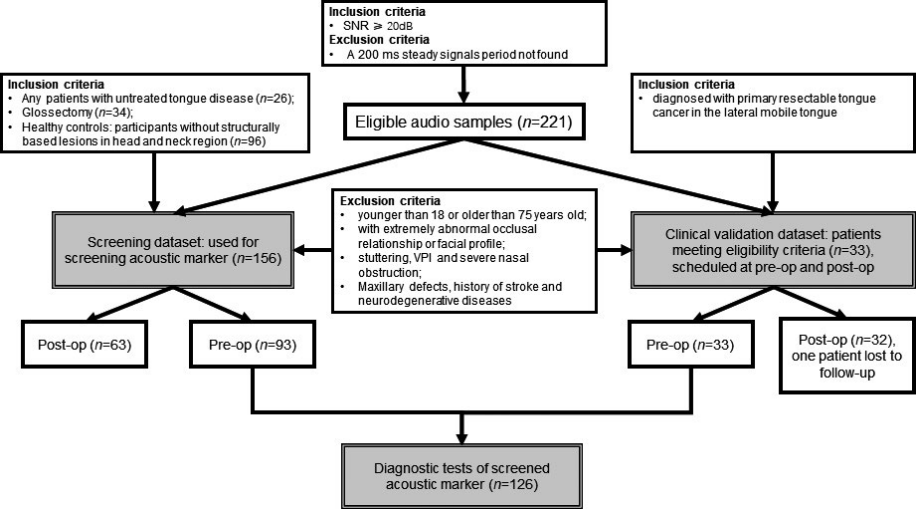
Flowchart in collection of participants and audio recordings. Totally, 221 audio samples were collected in this study. Of these, 156 samples constituted the screening dataset, and the rest of 65 samples contributing to the clinical validation dataset. Finally, 126 pre‐operative audio samples were combined to conduct the diagnostic tests using ROC analysis. The three intercorrelated dataset were highlighted in gray boxes. Abbreviations: SNR, signal‐to‐noise ratio, VPI, velopharyngeal insufficiency

A set of audio samples were collected from outpatient department of oral and maxillofacial surgery at Affiliated Hospital of Stomatology, Sun Yat‐sen University. We applied relatively lenient criteria when collecting the screening dataset because we aimed to uncover the linguistic implications of acoustic markers. Thus, we included (a) any patients with untreated tongue disease, (b) glossectomy, and (c) healthy controls without structurally based lesions in the head and neck region. We excluded individuals (a) younger than 18 or older than 75 years old, (b) with an extremely abnormal occlusal relationship or facial profile, (c) who stutter, have velopharyngeal insufficiency and severe nasal obstruction, and (d) have maxillary defects, history of stroke and neurodegenerative diseases. Patients returning for regular follow‐up appointments were recruited to record their vowel utterances and articulation status in a quiet environment. During this procedure, we paid more attention to articulation than disease status. Therefore, we included all audio recordings, regardless of potentially identical speaker at different time points (see Figure 1).

### 2.2 Patients contributing to clinical validation

Bearing in mind that different tongue cancer locations have variably influential patterns on articulation,^20^ strict eligibility criteria were applied to this recruitment (see Figure 1). Information retrieved about patients included age, gender, T classification (based on the 8th American Joint Committee on Cancer (AJCC) guideline),^21^ the extent of resection, reconstruction versus not, and the type of pedicle flap used for reconstruction (if any). Each patient was recruited prospectively and scheduled to receive APeQoL both before (pre‐op) and 4–6 weeks after (post‐op) surgery.

Although it was noticeable that the sample inclusion criteria are not exactly same among the discovery dataset, validation dataset and diagnostic test, what we mostly consider in the first stage was that the complexity of a dataset input to the support vector machine (SVM) models was favourable for robustness and generalization performance. So, in the discovery phase, we collected a general and complex dataset (e.g., patients with untreated tongue disease and glossectomy) which is more in line with the real world for the sake of clinical application in the context of preoperative appraisal and longitudinal follow‐up. After screening out a sensitive marker, we paid more attention to a specific disease (e.g., tongue cancers) because such a validation dataset was of utmost clinical importance due to the high incidence of tongue cancer and the leading role of tongue body for speech production. As for the diagnostic test, it further approved the clinical application from the view of diagnosis.

### 2.3 Perceptual evaluation (Pe)

A corpus of stimuli was meticulously designed according to the phonologic features of Mandarin Chinese. Monosyllables with CV or consonant–vowel–vowel (CVV) contexts were selected and consonant phonemes were all tongue‐dominant including alveolar (/d/, /t/, /n/, /l/), alveolo‐palatal (/j/, /q/, /x/), and velar (/g/, /k/) phonemes (see Table S2). Professional annotations of the international phonetic association (IPA) were avoided as most head and neck surgeons were unfamiliar with them.

Participants were asked to pronounce each syllable three times naturally with moderate volume and speed. The articulation of consonant phonemes was evaluated face‐to‐face by a professional phonetician. Responses were recorded as correct or deviated articulation at the individual‐level and percent consonant correct (PCC) at the group level.^22^ For statistical analysis, correct responses were coded as one and deviated responses were coded as zero.

### 2.4 Extraction of acoustic parameters (A)

Each participant was asked to pronounce three Chinese corner vowels (/ɑ/, /i/, and /u/) three times in a sustained way (i.e., no less than one second) with moderate volume. Simultaneous recordings were obtained in a quiet environment. Audio Analyzer (version 2.6, © Pawel Krzywdzinski) installed on an iPad® Mini2 was used for audio recording. The microphone was placed 8–10 centimeters from the right front of the speakers’ lips. The sampling ratio was set as 44100 Hz, and each sample was recorded at a 16‐bit resolution. All audio recordings were saved as WAV files. Unlike previous studies, we did not use professional recording hardware in order to maximize the potential telemedical applications of our results.^23^, ^24^

Anonymous audio recordings were imported into Praat software version 6.0.49 for Windows (Paul Boersma & David Weenink, Netherlands, 2018) with the first five formants and F0 superimposed on the narrowband spectrogram. Next, a 200‐ms steady signal period was manually framed to extract F1 and F2 via 512‐points Fast Fourier transform (FFT). This was implemented by a Praat script. The following derived formant metrics indicative of lingual mobility were then computed based on F1 and F2: formant centralization ratio (FCR), vowel space area (VSA), Joos‐VSA (the base 10 logarithm of VSA), compact‐diffuse (CD), and grave‐acute (GA) distinctive features. The physiological interpretations of these metrics are illustrated in Table S1. ^17^, ^25^

### 2.5 Speech‐related QoL assessment

The speech handicap index (SHI)^19^ was used to assess the pre and post‐operative speech function‐related QoL of participants involved in the clinical validation. The SHI consists of 30 items, 28 of which are equally divided into speech and psychosocial subdomains (see Table S3 for details).

### 2.6 Supervised machine learning

Using the screening acoustic marker dataset, the articulation outcomes of each monosyllable were used to label corresponding vowel formant metrics extracted and further computed from /ɑ/, /i/, and /u/ (as described above). A supervised binary classification was then performed using support vector machine (SVM) training models taking gender into account, giving the significant sexual dimorphism of vowel formant frequencies.^17^ The classification learner app within MATLAB 2018b (Mathworks, Natick, MA, USA) was employed for SVM classification and used to train classification models for each CV syllable. The cross‐validation was set as 10‐fold. Given that our primary aim was not to train a well‐generalized and robust model but to figure out which indicator was most important for classification outcome, the parameter settings did not undergo fine tuning but were preset as Table S4. Furthermore, the same formant metrics data structures from patients contributing to clinical validation data were inputted to the trained models to test their generalization property. In terms of machine learning, the dataset for screening marker and the dataset from patients contributing to clinical validation were the training and test sets, respectively.

To compare the relative significance of each vowel formant metric, the kernel function was fixed as linear in SVM. Consequently, the extent and direction of a corresponding parameter's influence on the classification outcomes could be quantified by each predictor's linear prediction coefficient (LPC).^26^

### 2.7 Statistical analyses

Two‐tailed Student's *t*‐tests were used to examine whether LPCs demonstrated significant sexual dimorphism. Mann–Whitney tests were used to test the sexual dimorphism of all formant metrics and SHI differences across articulation statuses. Cronbach's alpha, *α*, coefficient (obtained through reliability analyses) and Pearson correlation coefficient were used to test the internal consistency and content validity of SHI, respectively. One‐tailed Student's *t*‐tests were used to explore whether the sensitivity or sensitiveness of the acoustic marker differed significantly across articulation statuses based on the physiological interpretation. For trend analyses, we added a trend line to the scatter plot using regression analysis. The *R^2^* value was calculated to demonstrate the proportion of the variance in the data that was explained by the regression model. Two‐way analysis of variance (ANOVA) and mixed‐effects model analyses were used to investigate the influence of clinical variables on the screened acoustic marker. Chi‐square tests or Fisher's exact tests were used to analyze the distribution of parameters among the categorical clinical variables. Finally, we merged all of the pre‐operative audio recordings from both the training and test sets to investigate the screened acoustic marker's diagnostic power (in terms of T stages) using the receiver operating characteristic (ROC) curve. All analyses were conducted using Graphpad Prism 8.0.2 for Windows (GraphPad Software, San Diego, CA, USA) with the exception of the reliability analysis of SHI, which was performed in IBM SPSS Statistics for Windows, version 25.0 (IBM Corp., Armonk, NY, USA). If not stated otherwise, statistical significance is indicated as *(*p *< 0.05), **(*p* < 0.01), ***(*p* < 0.001), or *ns* (not significant).

## 3 RESULTS

### 3.1 Participants and audio samples

As shown in Figure 1, a total of 221 eligible audio samples were collected, of these, 156 audio (obtained from 80 males and 76 females) were used to screen the acoustic marker (i.e., as the screening dataset). The PCC analysis revealed that articulation status was almost identical across gender, but alveolo‐palatal consonants showed a predilection for misarticulation (Figure S1).

Under stricter eligibility criteria, 33 patients (20 males and 13 females) contributing to the clinical validation dataset. All of these patients were diagnosed with primary resectable tongue cancer in the lateral mobile tongue. Male and female patients were well matched in terms of age (*p* = 0.942), clinical T stage (*p* = 0.242), the extent of resection (*p* = 0.682), reconstruction or not (*p* = 0.182), and flap types used for reconstruction (*p *> 0.999) (Table 1). At post‐op, one male patient was lost to follow‐up. Therefore, a total of 65 audio samples formed the clinical validation dataset, and a follow‐up success rate of 97% was achieved.

**TABLE 1:** Clinical variables stratified by gender

Clinical variables	Male (*n *= 20)	Female (*n* = 13)	*p*
Age	52.15 ± 9.98	52.39 ± 7.84	*ns*
T classification^a^			
1	1 (5%)	3 (23.1%)	*ns*
2	7 (35%)	5 (38.5%)	
3	5 (25%)	1 (7.7%)	
4	7 (35%)	4 (30.8%)	
Extent of resection			
PG	6 (30%)	4 (30.8%)	*ns*
HG	9 (45%)	7 (53.8%)	
STG/TG	5 (25%)	2 (15.4%)	
Reconstruction			
Yes	18 (90%)	9 (69.2%)	*ns*
No	2 (10%)	4 (30.8%)	
Types of free flap			
ALT	15 (83.3%)	8 (88.89%)	*ns*
Others^b^	3 (16.7%)	1 (11.11%)	

Abbreviations: ALT, anterolateral thigh free flap; HG, hemiglossectomy; ns, not significant; PG, partial glossectomy; STG/TG, subtotal/total glossectomy.

^a^ According to the 8th AJCC guideline, T1 means tumor ≤ 2 cm and depth of invasion (DOI) ≤ 5 mm; T2 means tumor ≤ 2 cm, DOI >5 mm and ≤10 mm or tumor >2 cm but ≤4 cm, and DOI ≤10 mm; T3 means tumor >4 cm or any DOI >10 mm; T4 means moderately advanced or very advanced local disease.

^b^ Three male patients received reconstruction with pectoralis major musculocutaneous flap (PMMF), and bilobed radial forearm free flap (RFFF) for the female patient.

### 3.2 Indicative acoustic marker screened from SVM models

**FIGURE 2:**
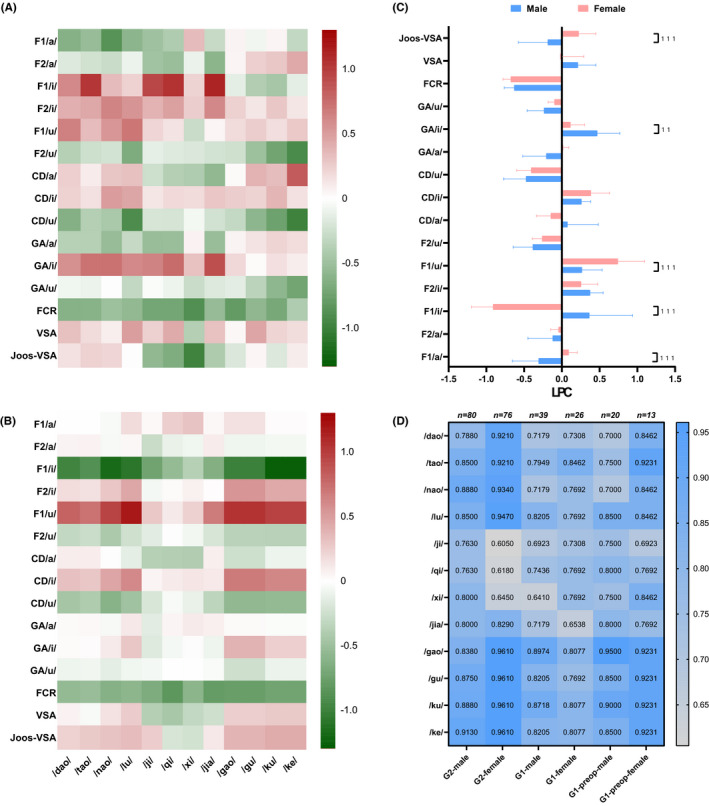
Each acoustic parameter's LPC values for each syllable‐specific model and generalization properties of SVM models. (A) The LPC heatmap of male participant, (B) the LPC heatmap of female participant, (C) the comparisons of average LPC values across gender via two‐tailed Student's *t*‐tests, and (D) the accuracies of trained SVM models and their generalization properties. Herein, we denoted clinical validation dataset as G1 and screening dataset as G2. Thus, G1‐pre‐op meant the pre‐operative data of clinical validation dataset

Since this study's primary aim was to screen an acoustic marker that was most closely correlated with the perceptual outcomes, we focused mainly on the LPCs of the SVM models. The models’ accuracy is displayed through a heatmap (Figure 2D), which showed an approximately numerical continuum with a descending order, where velar>alveolar>alveolo‐palatal. All models’ performance in terms of their training time, number of support vectors (#SV), area under curve (AUC), sensitivity, specificity, positive predictive value (PPV), and negative predictive value (NPV) was delineated in the Table S5. The trained SVM models were tested by the clinical validation datasets. The generalization properties were specific to only the pre‐operative and all of the data, respectively (Figure 2D).

The LPCs were extracted from all 24 SVM models and displayed as two gender‐specific heatmaps. The 15 rows and 12 columns represented 15 analyzed acoustic parameters and 12 syllables, respectively (Figure 2A and B). The FCR was the most dominant parameter in predicting perceptual outcomes. The values of the LPCs for FCR were all negative. This indicated that the larger the FCR, the greater the probability of misarticulation. There was no significant gender‐based difference between FCR’s average LPCs (*p* > 0.999) (Figure 2C). Although the significance of F1/i/ and F1/u/ was greater than FCR in the female group, significant sexual dimorphism of LPCs was found in F1/i/ (*p *< 0.001), F1/u/ (*p* < 0.001), F1/ɑ/ (*p* < 0.001), GA/i/ (*p* = 0.002), and Joos‐VSA (*p* < 0.001) (Figure 2C).

### 3.3 Correlation analysis within APeQoL

The pre‐operative data within the clinical validation dataset revealed that Cronbach's α coefficients for total, speech, and psychosocial domains were 0.974、0.938、and 0.954, respectively. Pearson correlation coefficients between the speech domain, the psychosocial domain, and SHI were 0.961 (*p* < 0.001), and 0.966 (*p* < 0.001), respectively.

**FIGURE 3:**
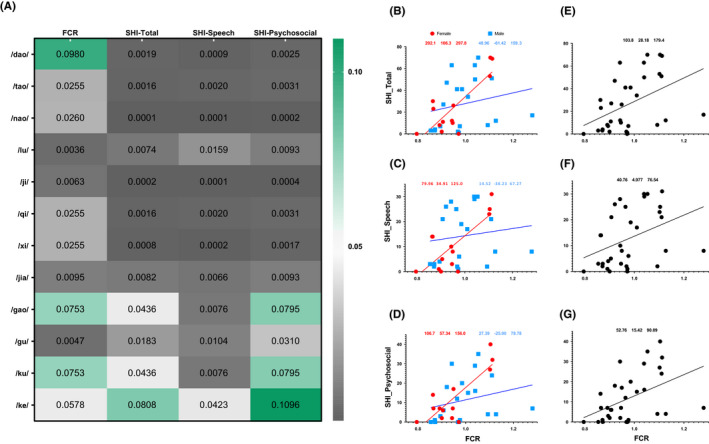
The consistency among perceptual outcome, FCR and SHI. (A) Heatmap for *p* values dictating the differences in FCR and SHI between correct and deviated perceptual outcomes via one‐tailed Student's *t*‐tests. (B, C, D) Gender‐stratified nonlinear fit between FCR and SHI (total, speech, and psychosocial domains) revealed that SHI‐total, SHI‐speech, and SHI‐psychosocial scores of female patients were all correlated significantly with FCR but none SHI scores of male patients were correlated significantly with FCR. (E, F, G) Gender‐combined non‐linear fit between FCR and SHI (total, speech, and psychosocial domains) revealed that SHI‐total, SHI‐speech, and SHI‐psychosocial scores were all correlated significantly with FCR. The numerical expressions on top of each figure from (B) to (G) represented the estimated slope (95% confidence interval)

One‐tailed Mann–Whitney tests conducted on gender‐combined data revealed that speech domain scores were all significantly higher for each syllable's deviated articulation. In contrast, total and psychosocial domain scores were only significantly higher for alveolar and alveolo‐palatal deviated outcomes. One‐tailed Student's *t*‐tests revealed that all deviated alveolo‐palatal perceptual outcomes had significantly higher FCR. Differences in FCR and SHI’s total, speech, and psychosocial scores for each syllable were visualized by a heatmap of *p* values (Figure 3A).

Gender‐combined data showed that SHI scores increased significantly with increasing FCRs (*p* = 0.009, *p* = 0.027, and *p *= 0.007 for total, speech, and psychosocial domains, respectively). Gender‐stratified analyses found that each SHI score in the female group responded significantly to FCRs (*p* < 0.001, *p* = 0.002, and *p *< 0.001 for total, speech, and psychosocial domains, respectively) (Figure 3B–G).

### 3.4 Preoperative and longitudinal analyzes of FCR

**FIGURE 4:**
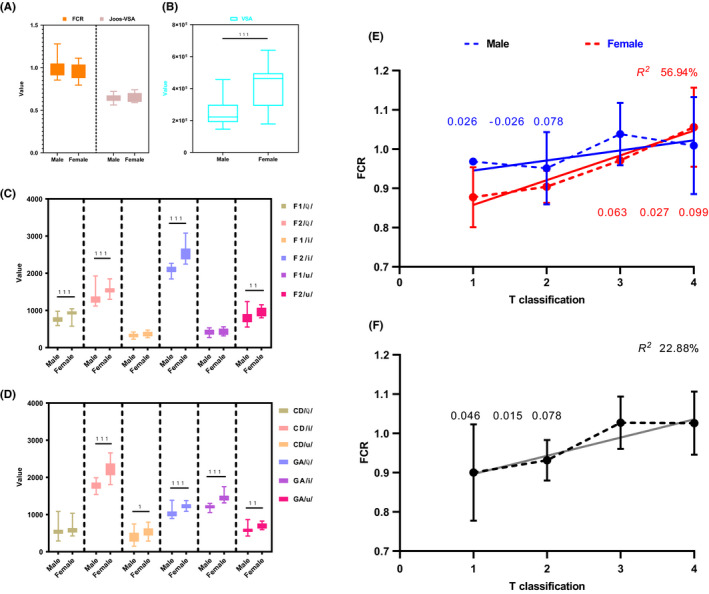
Differences of acoustic parameters across gender and pre‐operative trend analyses of FCR with regard to increasing T classification. (A, B, C, D) 1–99% percentile range of each vowel formant metric and their comparisons across gender via Mann–Whitney tests. (E) Gender‐stratified analysis of FCR by T classification revealed that the values of female patients responded significantly with an estimated slope 0.063 (95% confidence interval (CI): 0.027–0.099) and coefficients of determination (*R^2^*), 56.94%. (F) Gender‐combined analysis of FCR by T classification revealed that the values responded significantly with an estimated slope 0.046 (95% CI: 0.015–0.078) and *R^2^*, 22.88%

Significant gender differences were found for the majority of the vowel formant metrics (Figure 4A–D). Pre‐operatively, trend analyses for FCR by increasing T classification were performed on both gender‐stratified and gender‐combined datasets. It was found that FCR responded significantly to T classification with a slope of 0.063 (95% confidence interval (CI): 0.027–0.099, *p* = 0.003) and in female patients (Figure 4E) and with a slope of 0.046 (95% CI: 0.015–0.078; *p* = 0.005) in all patients (Figure 4F).

**FIGURE 5:**
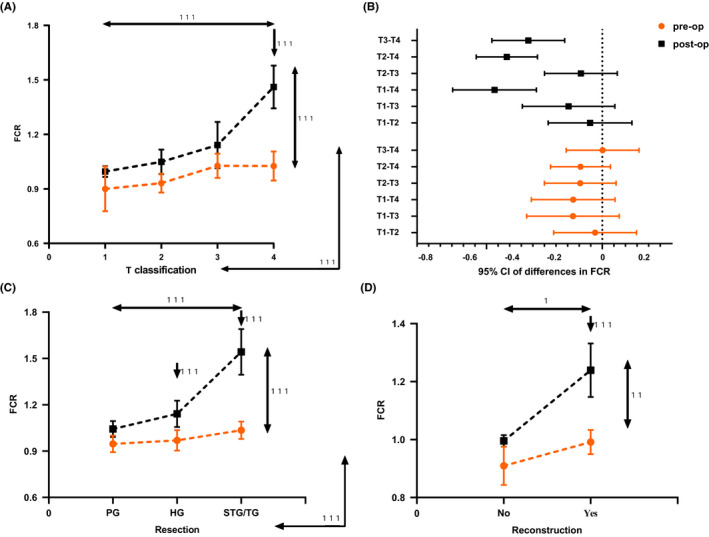
Comparisons between pre‐operative and post‐operative FCR with regard to clinical factors. (A, C, D) Mixed‐effect models to analyze the changes of FCR from pre‐operative to post‐operative status revealed that no matter what kind of clinical factors (i.e., T classification, the extent of resection, and reconstruction or not) as covariate, the main effects of time (pre‐op vs. post‐op) were significant, which were displayed via vertical two‐way arrows on the right of the figures. And the main effects of clinical factors were all significant for FCR, which were displayed via horizontal two‐way arrows on top of the figures. Interaction effects between clinical factors and time (pre‐op vs. post‐op) were all significant, which were displayed via perpendicularly crossing two‐way arrows on lower right corner of the figures. Further pairwise comparisons found that the FCR of T_4_, HG, STG/TG, and reconstruction groups increased significantly after surgery. (B) Multiple comparisons within pre‐operative or post‐operative FCR with regard to T classification revealed that only the differences between T_1_, T_2_, T_3_, and T_4_ after surgery were, respectively, of statistical significance at *p* < 0.05. Abbreviations: PG, partial glossectomy, HG, hemiglossectomy, STG/TG, subtotal/total glossectomy

Longitudinally, models with mixed effects revealed that clinical T classification (*p* < 0.001), the extent of resection (*p* < 0.001), and reconstruction or not (*p* = 0.013) all showed significant main effects on FCR. Interaction effects between the clinical T classification (*p* < 0.001), resection (*p* < 0.001), and time (pre‐op vs. post‐op) were all significant on FCR (Figure 5A–D). Further pairwise comparisons found that the FCRs of the T_4_ (*p* < 0.001), hemiglossectomy (*p* < 0.001), subtotal/total glossectomy (*p* < 0.001), and reconstruction (*p* <0.001) groups increased significantly after surgery (Figure 5A–D).

### 3.5 Preoperative and longitudinal analyses of SHI

**FIGURE 6:**
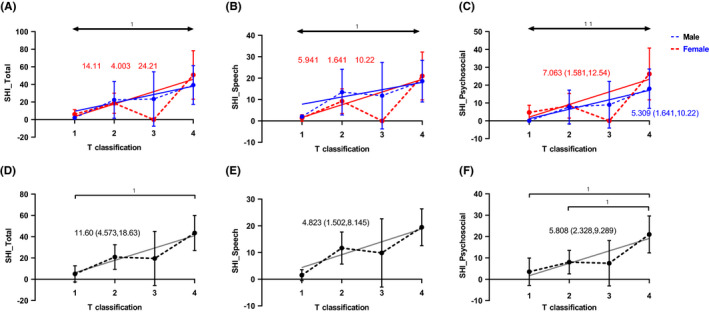
Pre‐operative analysis of SHI with regard to T classification. (A, B, C). Pre‐operative gender‐stratified analyses of SHI with regard to T classification via two‐way ANOVA and non‐linear fit revealed the significant main effects of T classification on SHI scores in the female subgroup. The SHI responded significantly with an estimated slope 14.11 (95% CI, 4.003–24.21), 5.941 (95% CI, 1.641–10.22), and 7.063(95% CI, 1.581–12.54) for total, speech, and psychosocial domains, respectively. Only psychosocial domain in male subgroup responded significantly to T classification with an estimated slope 5.309 (95% CI, 1.641–10.22). The horizontal two‐way arrows on top of the figures represented the main effects of T classification. (D, E, F) Pre‐operative gender‐combined analysis of SHI with regard to T classification via nonlinear fit revealed that SHI scores responded significantly with an estimated slope 11.60 (95% CI, 4.573–18.63), 4.823 (95% CI, 1.502–8.145), and 5.808 (95% CI, 2.328–9.289) for total, speech, and psychosocial domains, respectively

Pre‐operatively, T classification had a significant main effect on SHI total scores (*p* = 0.014) and sub‐dimensional scores (*p* = 0.034 for the speech domain and *p* = 0.007 for the psychosocial domain), which were not affected by gender or gender‐T classification interactions (Figure 6A–C). Gender‐combined outcomes showed that SHI scores responded significantly to T classification (*p* = 0.002, *p* = 0.006, and *p* = 0.002 for total, speech, and psychosocial domains, respectively) (See Figure 6D–F).

Longitudinally, all of the main effects of time (pre‐op vs. post‐op) on SHI scores were significant. In contrast, all clinical variables (i.e., T classification, resection, and reconstruction) only displayed consistent main effects on the speech domain. The interaction effect between time and T classification was only significant for the speech domain. Surprisingly, patients with T_3_ had significantly higher total, speech, and psychosocial scores after the surgery (See Table S6).

### 3.6 Diagnostic power of FCR

**FIGURE 7:**
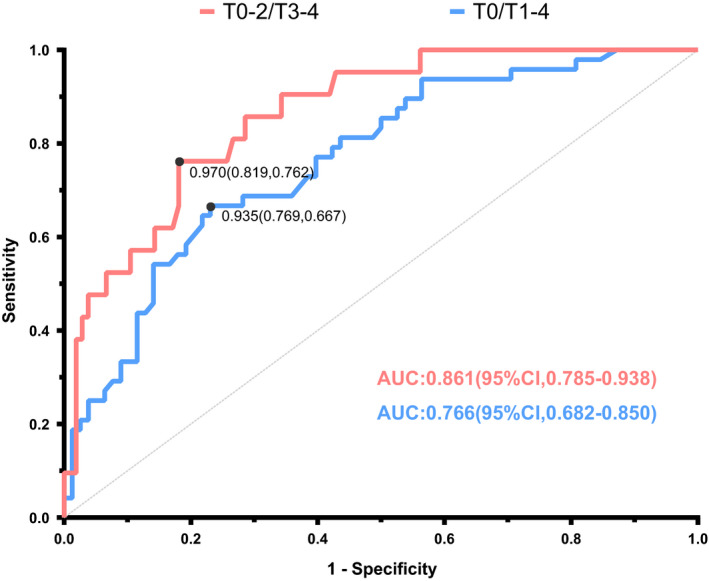
ROC curves of FCR in discriminating different T status. After merging the pre‐operative data from screening and clinical validation datasets, 126 individual cases were obtained. In discriminating T_0‐2_ from T_3‐4_, a cut‐off value of 0.970 produced an 81.9% specificity and a 76.2% sensitivity, and the AUC was 0.861 (95% CI: 0.785–0.938; *p* < 0.001). In discriminating T_0_ (healthy controls) from T_1‐4_ (tongue cancer patients), a cut‐off value of 0.935 produced a 76.9% specificity and a 66.7% sensitivity, and the AUC was 0.766 (95% CI: 0.682–0.850, *p* < 0.001). Abbreviation: AUC, area under curve

After combining all of the pre‐operative data in both screening and clinical validation datasets, 126 participants were included in the ROC analysis. This analysis revealed two optimal cut‐off values for FCR in discriminating T_0‐2_ from T_3‐4_ and T_0_ from T_1‐4_ (Figure 7). A cut‐off value of 0.970 produced an AUC of 0.861 (95% CI: 0.785–0.938; *p *< 0.001) for T_3‐4_ patients with a 76% sensitivity and an 82% specificity.

## 4 DISCUSSION

This study investigated a novel method for screening an acoustic marker for tongue cancers and performed a clinical validation of its findings. In the screening phase, we screened out the FCR as a characteristic marker using a data‐driven approach with SVM. Thereafter, we prospectively collected a dataset from prescheduled tongue cancers to validate the FCR’s discriminative ability in the preoperative appraisal and its evaluative property under the circumstance of longitudinal follow‐up. Our results primarily indicated that FCR is an acoustic marker with the potential to detect disease and related speech function in oral tongue cancers.

Despite our first attempt to classify the acoustic parameters extracted from three vowels /ɑ/, /i/, and /u/, the dichotomous articulation status of several tongue‐dominant consonants indicated that our findings were generally consistent with other results obtained through electropalatographic analysis,^27^ whose phonetic explanation was beyond the scope of this clinical research. Our study relied on monosyllabic articulation status rather than comprehensive speech function as previous studies reported that PCC was more sensitive for assessing the speech function of patients with oral and oropharyngeal cancers.^22^, ^28^, ^29^ Furthermore, the stimuli used for Pe in this study were specifically chosen based on the phonological rules of Mandarin Chinese and can be adjusted to several Chinese dialects.

### 4.1 Speech disorder of tongue cancer patients

Currently, there is a lack of large‐scale epidemiological studies on the speech disorders in tongue cancer patients. This may have two underlying explanations. First, few‐to‐no evident speech disorders manifest during the early stages of the disease, resulting in a lack of consultations to speech‐language pathologists (SLPs). Second, the mutual cooperation between head and neck surgeons and SLPs demands an in‐depth framework to attract more attention from doctors and patients. However, Wang et al. conducted a SEER‐based analysis of rehabilitation services utilization in 16194 patients with HNC in the United States.^30^ They found that the overall utilization rate was 20.7% for SLP and 26.2% for occupational/physical therapy services.^30^

Colangelo et al. investigated the pretreatment relation between tumor burden and speech and swallowing function in 230 patients with oral or oropharyngeal cancer prior to surgery.^12^ Of these patients, only 62 had tongue cancer. In terms of consonant phonemes, the palato‐alveolar phonemes /ʃ/、/ʒ/、/ʧ/、/ʤ/ (which are all fricatives or affricates) were more likely to be mispronounced. Similarly, the alveolo‐palatal phonemes /j/, /q/, /x/ had the lowest PCC in our current study (see Figure S1). Studies of other diseases or disorders that manifest evident speech disorders (such as Prader‐Willis Syndrome) have also found that palato‐alveolar phonemes were much easier to mispronounce than other phonemes.^31^ When considering the physiological mechanism embedded in articulation, the pronunciation of palato‐alveolar or alveolo‐palatal phonemes requires more complicated coordination of speech.^18^

### 4.2 Enlightenment of the LPC on individual speech rehabilitation

The LPC was a distinct coefficient in the SVM algorithm when the kernel function was linear. This indicated that the original data were linearly scaled. Thus, we were able to state that the larger value of a parameter's LPC, the more significant role for the articulation. We later clinically validated the meanings of LPC based on finding that FCR was a sensitive parameter to disease status and related treatments. The FCR, calculated based on F1 and F2 of three corner vowels (/ɑ/, /i/, and /u/, see Table S2), was first introduced by Sapir et al.^32^ as a novel acoustic measure of dysarthric speech secondary to idiopathic Parkinson's disease. Moreover, the LPC matrix may underlie constructive implications for individual speech rehabilitation. The considerable importance of FCR for articulation provided acoustic insights for the specific practice of complex tasks as a whole rather than practice of its simpler components,^33^ which also aligned with the theory of motor learning.^29^, ^34^ Nevertheless, for some acoustic parameters (e.g., F1/i/ and F1/u/) the absolute LPC values were bigger than the FCR’s (see Figure 1A and B). This suggested that non‐specific exercises in speech rehabilitation, such as tongue range of mobility exercises, may outperform targeted exercises for some consonant phonemes, as reported by previous studies.^35^ Implementing precise speech rehabilitation for patients with tongue cancers and other HNC must incorporate the individual and phonemic level.

### 4.3 Clinical implication of FCR

The FCR was generally consistent with other Pe and QoL assessments. From the perspective of holism, there should be a consistency among all assessment methods applied to individuals. However, When Dwivedi et al. ^36^ investigated the acoustic parameters of speech and their correlation with QoL and Pe in patients with oral cavity and oropharyngeal cancer, they did not establish consistency between SHI, speech assessment outcome, and formant frequencies measurements. One evident shortcoming of their research was that they only included the F0, F1 and F2 of sustained vowel /i/. It has been widely reported that F1 and F2 were not as reliable and relevant as other measurements, irrespective of their well‐established physiological interpretation.^37^, ^38^ Most importantly, formant frequencies used in previous studies were primarily restricted to their original aspects and not extended to their derived and comprehensive properties. As FCR is a derived and comprehensive parameter of more dynamic connotations, it maps well to pathological speech‐language status. Since its discovery, FCR has demonstrated strong discriminative properties for speech disorders, not limited to dysarthria. Functional articulation disorder^39^ and speech disorder related to hearing impairments^40^ could also be detected and quantified by FCR. To the best of our knowledge, our study was the first to extend the application of FCR to the structurally based articulation disorder resulting from tongue cancer. Moreover, we clinically validated FCR as a potential parameter for the automatic detection of clinical T classification of tongue cancer and especially for discriminating T_0‐2_ from T_3‐4_ patients. We demonstrated the pre‐operative discriminative competence and longitudinal evaluative strength of FCR. Similarly, Sauvageau et al. found that FCR could be used to detect changes before and after deep brain stimulation of the subthalamic nucleus and levodopa intake in Parkinson's disease by.^41^ Our study extended the potential application of FCR to the automatic detection of tongue cancers not only in a traditional clinical setting, but also via telemedical given our crude recording equipment and environments.

### 4.4 Concerns with speech‐related QoL

SHI is a universally adopted questionnaire used to assess the speech‐related QoL. Our studies and previous studies have established its reliability and validity.^42^, ^43^, ^44^, ^45^, ^46^ Specifically, our results demonstrated that T_3_ patients’ speech‐related QoL worsened significantly after surgery (see Table S4). According to the 8th AJCC guidelines, T_3_ tumors are still encapsulated within the internal lingual muscles. The typical surgical treatment used in this subgroup may explain the drastic increase in SHI scores. Specifically, extended tumor resection inevitably causes substantial loss of external lingual muscles and seriously comprises overall tongue mobility. Thus, T_3_ patients may require timely psychological supports and intense speech rehabilitation.

### 4.5 Limitations

This study has several limitations that warrant mention. Our objective acoustic parameters were not comprehensive in terms of phonetic description because we only analyzed the vowel formants and their derived measurements. Since we focused on a single articulator (i.e., the tongue), parameters corresponded to the status of vocal folds (e.g., F0, jitter, and shimmer) were excluded from analysis.^13^ The small sample size and short‐term follow‐up provide ample opportunities for subsequent studies, such as those interested in investigating the impacts of radiotherapy and chemotherapy. Finally, the preliminary results suggest that FCR may be a discriminative and evaluative marker of oral tongue cancers. These results need to be replicated through longitudinal studies and/or in larger cohorts.

## 5 CONCLUSION

This study applied APeQoL to assess the speech profiles of patients with tongue cancer in regard to T classification, the extent of resection, and reconstruction. It determined that FCR may be an indicative acoustic marker of both discriminative and evaluative speech properties, independent of the innate sexual dimorphism of formant frequencies. The methodology also provided novel insights for individual speech rehabilitation.

## CONFLICTS OF INTERESTS

The authors declare that they have no conflict of interest.

## ETHICS STATEMENT

All procedures performed in this study involving human participants were in accordance with the ethical standards of the institutional and national research committee and with the 1964 Helsinki declaration and its later amendments or comparable ethical standards. The protocol was approved by the Ethical Committee of Affiliated Hospital of Stomatology at Sun Yat‐Sen University. Written informed consent was obtained from each participant.

## Supporting information

Figure S1

Table S1‐S6

## Data Availability

The raw data supporting the conclusions of this manuscript will be made available by the authors, without undue reservation, to any qualified researcher.
