# Supplementary material for: Data mining of an acoustic biomarker in tongue cancers and its clinical validation
Source: Cancer Med. 2021 May 2;10(11):3822–35. doi: 10.1002/cam4.3872 (PMC8178493; doi:10.1002/cam4.3872)
Supplement: Supplementary file 1 — Figure S1 [file CAM4-10-3822-s001.docx]

**Supplements for manuscript**

*Data Mining of an Acoustic Biomarker in Tongue Cancers and its Clinical Validation*

**
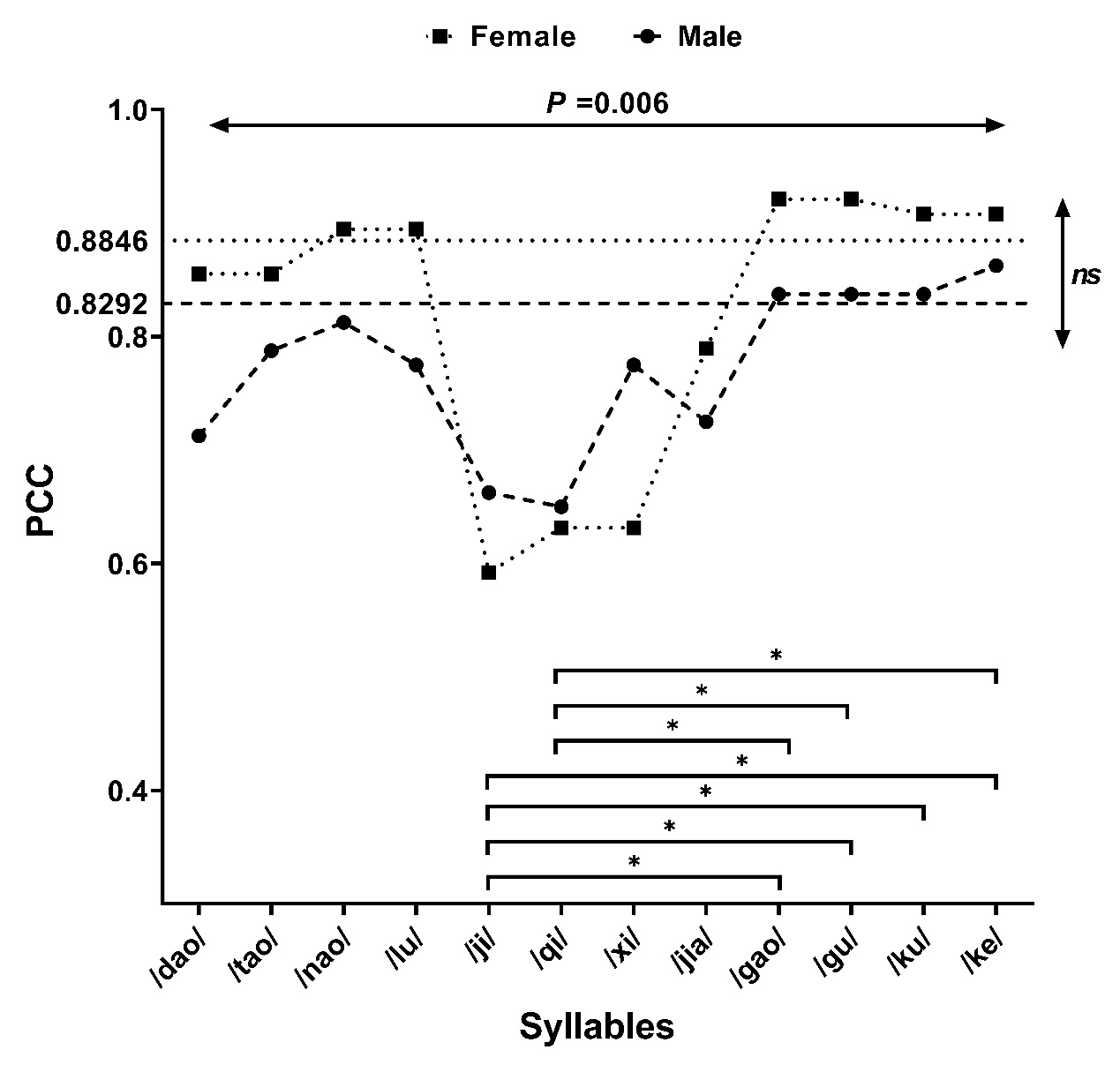
**

**Supplementary. Figure 1 PCC analysis with regard to syllables and gender**

Two-way ANOVA revealed the main effect of syllables was significant (*P* =0.006) but not for gender. Mean PCCs were 88.46% and 82.92% for female and male respectively. Pairwise comparisons found the PCC of /ji/ was significantly lower than those of all velar syllables and PCC of /qi/ was significantly lower than those of all velar syllables except for /ku/.

Abbreviations: PCC = percent consonant correct, ns = not significant.
