# Supplementary material for: Data mining of an acoustic biomarker in tongue cancers and its clinical validation
Source: Cancer Med. 2021 May 2;10(11):3822–35. doi: 10.1002/cam4.3872 (PMC8178493; doi:10.1002/cam4.3872)
Supplement: Supplementary file 2 — Table S1‐S6 [file CAM4-10-3822-s002.docx]

**Supplements for manuscript**

*Data Mining of an Acoustic Biomarker in Tongue Cancers and its Clinical Validation*

**Supplementary. Table 1** The analyzed acoustic feature sets indicating tongue position and mobility

| Vowel formant metrics | Extraction Methods or Formula | Physiological interpretation | |
| --- | --- | --- | --- |
| F1 | 512-point FFT for a steady vowel utterance of 200ms | an index of the height of tongue position | Bigger value indicates lower height, and vice versa. |
| F2 | Same as F1 | an index of the anteroposterior movement of tongue | Bigger value indicates more fronting of tongue, and vice versa. |
| FCR | (F2/u/+F2/ɑ/+F1/i/+F1/u/) ÷ (F2/i/+F1/ɑ/) | an index of vowel centralization | Bigger value indicates poor articulation. |
| VSA | 0.5×[(F2/u/+F2/i/)×(F1/u/−F1/i/)–(F2/ɑ/ +F2/u/)×(F1/u/−F1/ɑ/)–(F2/ɑ/+F2/i/)×(F1/ɑ/−F1/i/)] | an index of formant frequency ranges | Bigger value generally indicates better articulation. |
| Joos-VSA | the base 10 logarithm of VSA | Same as VSA | Same as VSA |
| CD | F2 – F1 | an index of tongue elevation | Bigger value indicates more tongue elevation but not necessarily for /u/, and vice versa. |
| GA | (F1 + F2)/2. | an index of tongue advancement | Bigger value indicates more tongue advancement, and vice versa. |

Abbreviations: F1=first formant, F2=second formant, FCR=formant centralization ratio, VSA=vowel space area, CD= compact-diffuse distinctive feature, GA= grave-acute distinctive feature, FFT=fast Fourier transform.

**Supplementary. Table 2** The corpus of syllables used for perceptual evaluation

| Consonant phonemes | | Syllables | Manners of articulation |
| --- | --- | --- | --- |
| alveolar | /d/ | /dao/ | Stop |
|  | /t/ | /tao/ | Stop |
|  | /n/ | /nao/ | Nasal |
|  | /l/ | /lu/ | Lateral approximant |
| Alveolo-palatal | /j/ | /ji/, /jia/ | Affricate |
|  | /q/ | /qi/ | Affricate |
|  | /x/ | /xi/ | Fricative |
| velar | /g/ | /gao/, /gu/ | Stop |
|  | /k/ | /ku/. /ke/ | Stop |

**Supplementary. Table 3** Speech Handicap Index (SHI)

0 = Never 1 = Almost Never 2 = Sometimes 3 = Almost Always 4 = Always

| 1. My speech makes it difficult for people to understand me | 0 | 1 | 2 | 3 | 4 |
| --- | --- | --- | --- | --- | --- |
| 2. I run out of air when I speak | 0 | 1 | 2 | 3 | 4 |
| 3. The intelligibility of my speech varies throughout the day | 0 | 1 | 2 | 3 | 4 |
| 4. My speech makes me feel incompetent | 0 | 1 | 2 | 3 | 4 |
| 5. People ask me why I am hard to understand | 0 | 1 | 2 | 3 | 4 |
| 6. I feel annoyed when people ask me to repeat | 0 | 1 | 2 | 3 | 4 |
| 7. I avoid using the phone | 0 | 1 | 2 | 3 | 4 |
| 8. I am tense when talking to others because of my speech | 0 | 1 | 2 | 3 | 4 |
| 9. My articulation is unclear | 0 | 1 | 2 | 3 | 4 |
| 10. People have difficulty understanding me in a noisy room | 0 | 1 | 2 | 3 | 4 |
| 11. I tend to avoid groups of people because of my speech | 0 | 1 | 2 | 3 | 4 |
| 12. People seem irritated with my speech | 0 | 1 | 2 | 3 | 4 |
| 13. People ask me to repeat myself when speaking face-to-face | 0 | 1 | 2 | 3 | 4 |
| 14. I speak with friends and neighbors or relatives less often because of my speech | 0 | 1 | 2 | 3 | 4 |
| 15. I feel as though I have to strain to speak | 0 | 1 | 2 | 3 | 4 |
| 16. I find other people do not understand my speaking problem | 0 | 1 | 2 | 3 | 4 |
| 17. My speaking difficulties restrict my personal and social life | 0 | 1 | 2 | 3 | 4 |
| 18. The intelligibility is unpredictable | 0 | 1 | 2 | 3 | 4 |
| 19. I feel left out of conversations because of my speech | 0 | 1 | 2 | 3 | 4 |
| 20. I use a great deal of effort to speak | 0 | 1 | 2 | 3 | 4 |
| 21. My speech is worse in the evening | 0 | 1 | 2 | 3 | 4 |
| 22. My speech problem causes me to lose income* | 0 | 1 | 2 | 3 | 4 |
| 23. I try to change my speech to sound different* | 0 | 1 | 2 | 3 | 4 |
| 24. My speech problem upsets me | 0 | 1 | 2 | 3 | 4 |
| 25. I am less outgoing because of my speech problem | 0 | 1 | 2 | 3 | 4 |
| 26. My family has difficulty understanding me when I call them throughout the house | 0 | 1 | 2 | 3 | 4 |
| 27. My speech makes me feel handicapped | 0 | 1 | 2 | 3 | 4 |
| 28. I have difficulties to continue a conversation because of my speech | 0 | 1 | 2 | 3 | 4 |
| 29.I feel embarrassed when people ask me to repeat | 0 | 1 | 2 | 3 | 4 |
| 30. I am ashamed of my speech problem | 0 | 1 | 2 | 3 | 4 |

The total scores of SHI range from 0 to 120. Speech domain covers a total of 14 items: 1,2,3,5,6,9,10,13,15,18,20,21,26,28, which result in a maximal score of 56. Psychosocial domain covers a total of 14 items: 4,7,8,11,12,14,16,17,19,24,25,27,29,30, which also result in a theoretical maximum of 56.

**Supplementary. Table 4** Preset SVM hyperparameters

| Parameters | Settings |
| --- | --- |
| Cross-validation | 10-fold |
| Regularization parameter (C) | 1 |
| Kernel function | Linear |
| Kernel scale | Automatic |
| Solver | SMO |
| Standardized data | True |
| PCA | Disabled |

Abbreviations: SVM= support vector machine, SMO= sequential minimum optimization, PCA= principal component analysis

**Supplementary. Table 5** SVM models’ performance

| SVM  models | | Training Time | #SV | AUC | Sensitivity | Specificity | PPV | NPV |
| --- | --- | --- | --- | --- | --- | --- | --- | --- |
| Female  (*n*=76) | Dao | 0.62955 | 20 | 0.84 | 45% | 100% | 100% | 92% |
|  | Tao | 0.62729 | 19 | 0.83 | 45% | 100% | 100% | 92% |
|  | Nao | 0.82783 | 16 | 0.90 | 38% | 100% | 100% | 93% |
|  | Lu | 0.7416 | 14 | 0.96 | 50% | 100% | 100% | 94% |
|  | Ji | 0.62156 | 59 | 0.52 | 23% | 87% | 54% | 62% |
|  | Qi | 0.63954 | 55 | 0.55 | 21% | 85% | 46% | 65% |
|  | Xi | 0.78958 | 55 | 0.60 | 18% | 92% | 56% | 66% |
|  | Jia | 0.66049 | 33 | 0.71 | 19% | 100% | 100% | 82% |
|  | Gao | 0.66905 | 13 | 1 | 50% | 100% | 100% | 96% |
|  | Gu | 0.79419 | 13 | 1 | 50% | 100% | 100 | 96% |
|  | Ku | 0.68355 | 13 | 0.95 | 57% | 100% | 100% | 96% |
|  | Ke | 0.6288 | 13 | 0.96 | 57% | 100% | 100% | 96% |
| Male  (*n*=80) | Dao | 0.63722 | 40 | 0.81 | 48% | 91% | 69% | 81% |
|  | Tao | 0.69271 | 33 | 0.78 | 47% | 92% | 62% | 87% |
|  | Nao | 0.75575 | 28 | 0.85 | 47% | 97% | 78% | 89% |
|  | Lu | 0.67661 | 31 | 0.89 | 50% | 92% | 64% | 86% |
|  | Ji | 0.57595 | 51 | 0.70 | 41% | 91% | 69% | 75% |
|  | Qi | 0.7173 | 49 | 0.70 | 43% | 90% | 71% | 75% |
|  | Xi | 0.7635 | 38 | 0.66 | 6% | 95% | 25% | 78% |
|  | Jia | 0.54417 | 38 | 0.79 | 50% | 93% | 73% | 83% |
|  | Gao | 0.56456 | 29 | 0.65 | 15% | 100% | 100% | 86% |
|  | Gu | 0.5481 | 26 | 0.78 | 31% | 99% | 80% | 88% |
|  | Ku | 0.75416 | 24 | 0.84 | 46% | 97% | 75% | 90% |
|  | Ke | 0.9367 | 20 | 0.91 | 45% | 99% | 83 | 92% |

Abbreviations: SVM= support vector machine, #SV= number of support vectors, AUC= area under curve, PPV= positive predictive value, NPV= negative predictive value

**Supplementary. Table 6** Longitudinal analysis of SHI with regard to clinical variables

| **SHI domains** | | | **T-pre**  **(mean)** | **T-post**  **(mean)** | **Within-group**  **differences(*P*)** | **Main effects** | | **Interaction effects** |
| --- | --- | --- | --- | --- | --- | --- | --- | --- |
|  |  |  |  |  |  | **Time** | **Clinical factors** |  |
| **Total** | **T classification** | T_1_ | 5 | 24.75 | 0.490 | <0.0001*** | 0.003** | 0.152 |
|  |  | T_2_ | 20.83 | 39.35 | 0.181 |  |  |  |
|  |  | T_3_ | 19.50 | 71.18 | 0.002** |  |  |  |
|  |  | T_4_ | 43.45 | 61.45 | 0.182 |  |  |  |
|  | **Resection** | PG | 13 | 45.05 | 0.011* | <0.001*** | <0.001** | 0.632 |
|  |  | HG | 22.13 | 43.54 | 0.023* |  |  |  |
|  |  | STG/TG | 54.29 | 73.56 | 0.311 |  |  |  |
|  | **Reconstruction** | No | 12.17 | 34.61 | 0.152 | 0.002** | 0.061 | 0.883 |
|  |  | Yes | 29.33 | 53.79 | <0.001*** |  |  |  |
| **Speech** | **T classification** | T_1_ | 1.5 | 11.75 | 0.389 | <0.0001*** | <0.001*** | 0.045* |
|  |  | T_2_ | 11.67 | 19.59 | 0.241 |  |  |  |
|  |  | T_3_ | 9.83 | 38.68 | <0.001*** |  |  |  |
|  |  | T_4_ | 19.45 | 30.24 | 0.053 |  |  |  |
|  | **Resection** | PG | 6.9 | 21.2 | 0.023* | <0.001*** | 0.002** | 0.713 |
|  |  | HG | 10.81 | 24.09 | 0.004** |  |  |  |
|  |  | STG/TG | 25.29 | 33.64 | 0.417 |  |  |  |
|  | **Reconstruction** | No | 5.83 | 15.85 | 0.200 | 0.002** | 0.017* | 0.604 |
|  |  | Yes | 14.22 | 27.72 | <0.001*** |  |  |  |
| **Psychosocial** | **T classification** | T_1_ | 3.5 | 12.25 | 0.632 | <0.001*** | 0.017* | 0.313 |
|  |  | T_2_ | 8 | 17.08 | 0.227 |  |  |  |
|  |  | T_3_ | 7.5 | 28.87 | 0.014* |  |  |  |
|  |  | T_4_ | 21 | 27.33 | 0.529 |  |  |  |
|  | **Resection** | PG | 5.4 | 21.51 | 0.010* | <0.001*** | 0.001** | 0.364 |
|  |  | HG | 9.81 | 17.06 | 0.162 |  |  |  |
|  |  | STG/TG | 25 | 34.66 | 0.301 |  |  |  |
|  | **Reconstruction** | No | 6.17 | 16.79 | 0.185 | 0.006** | 0.204 | 0.926 |
|  |  | Yes | 12.93 | 22.91 | 0.005** |  |  |  |

Abbreviations: PG=partial glossectomy, HG=hemiglossectomy, STG/TG= subtotal/total glossectomy
